# Supplementary material for: Rural prioritization may increase the impact of COVID-19 vaccines in a representative COVAX AMC country setting due to ongoing internal migration: A modeling study
Source: PLOS Glob Public Health. 2022 Jan 27;2(1):e0000053. doi: 10.1371/journal.pgph.0000053 (PMC10021691; doi:10.1371/journal.pgph.0000053)
Supplement: Attachment — Submitted filename: DTK_Spatial_vaccine_distribution_response.pdf [file pgph.0000053.s002.pdf]

We thank the editor and the two reviewers for their feedback on our manuscript, *Rural prioritization may increase the impact of COVID-19 vaccination in COVAX AMC countries due to ongoing mobility: A modeling study*, for consideration for publication in *PLoS Global Public Health*. We respond to each of the reviewer comments below.

Reviewer #1

Dear Authors, I have reviewed the paper very carefully. This paper is very well-written and lots of novel analysis are there. Your manuscript proposed a lot of information on the dynamics of COVID-19. Just few minor changes are required. I suggest the followings:

1. Some typo mistakes are present in the manuscript. Please look into the paper carefully and correct them.
   We have corrected typos.

2. Cite the following papers:
   Kumar, P., Erturk, V. S., & Murillo-Arcila, M. (2021). A new fractional mathematical modelling of COVID-19 with the availability of vaccine. Results in Physics, 24, 104213.
   Kumar, P., Erturk, V. S., Murillo-Arcila, M., Banerjee, R., & Manickam, A. (2021). A case study of 2019-nCOV cases in Argentina with the real data based on daily cases from March 03, 2020 to March 29, 2021 using classical and fractional derivatives. Advances in Difference Equations, 2021(1), 1-21.
   We thank the reviewer for the kind comments and suggested citations. We added a citation to the first paper relating to vaccination in the Discussion.

Reviewer #2

The paper considers the effect of prioritizing urban or rural communities in the distribution of COVID-19 vaccines in averting infection within the Sub-Sahara African (SSA) region. The study uses an agent-based epidemiological model as implemented in Epidemiological MODeling software (EMOD). The paper is well designed and structured. The simulations are well thought, and good number of scenarios are analysed before coming to conclusion. The analysis and conclusion of the paper are interesting and practical. However, the following are some of the shortcomings identified by this reviewer.

1. In adapting the software EMOD, the authors did not indicate that they have considered and implemented in the model the difference in the epidemiological behaviour of the Symptomatic and Asymptomatically infectious individuals. It is natural to assume that the contact rates between individuals with symptomatic and asymptomatic persons vary significantly. It is reported in many of the epidemiological literature that most young individuals are observed not to show the symptoms of the disease. If this is true, it might significantly affect the dynamics of the disease in the society where a large proportion of its population is younger in age, like in the case of SSA region.
   We thank the reviewer for pointing out possible differences in younger populations that may have more asymptomatic infections. We now explain our assumption of treating both symptomatic and asymptomatic individuals as a single group in the Methods section, ll. 103ff. Briefly, we point out studies that have found viral load profiles to be similar between symptomatic and asymptomatic individuals. We also mention that the empirical evidence that infected individuals curtail their contact after symptom onset is lacking for LMICs, particularly for the majority of infections which do not require hospitalization.

2. When an NPI (specifically a lockdown rule) is introduced in many of the countries in the region, it has been observed that a large proportion of people travelled from the urban areas into rural areas before the rule is imposed. The model seems to not consider this fact into account.
   We thank the reviewer for pointing out possible confusion over how we have chosen to represent post-lockdown behavior. The scenarios we have chosen cover a range of migration possibilities including both higher and lower urban-to-rural migration that persist for the duration of the model. Because all scenarios begin with the appearance of the first case in the country at time 0, a higher migration rate approximates increased

urban-to-rural migration early in the pandemic. While we don't explicitly model a short duration uptick in migration, we now clarify that the high-migration scenario approximates an increase in reverse migration rates in the Results ll. 263ff. and Discussion ll. 424ff.

3. In the consideration of immigration, the direction of movement is not clearly indicated in the manuscript. If the direction is from the rural areas to urban ones, the direction is not always the same. Specially, the movement of the younger individuals depends on the time of the year and the need for the workforce in the economic activities. It is not clear to the reviewer how this situation is handled in the simulation.
We thank the reviewer for perceptively pointing out that the net direction and rate of migration may differ by season. In our model we focus on short-term bidirectional movement occurring on the timescale of COVID incubation and infectious periods. We do not explicitly model longer timescale seasonal migration that result in sub-annual net increases in either urban or rural populations; however, the impact of longer-timescale migration is captured in the population split between urban and rural settings in the model. We now clarify these points in the Methods (l. 72 and 80ff.) and Discussion. In addition, seasonal patterns of migration may differ between countries in SSA and would best be addressed by country-specific models which we also now indicate in the Discussion (l. 476, 508).

4. The final recommendation is based on the baseline scenarios described on pages 14 – 18. Except for the low migration scenario, all of them imply that the total number of infections in rural areas outnumber those in the urban areas. However, as a person experiencing the situation from within the region this reviewer can witness that, this does not reflect the reality on the ground. The infection is still very rare in most of the rural areas in the region. The authors need to check their assumptions to the data on the ground. On the other hand, authors compared their results on page 18 (line numbers 258 – 262) as consistent with the one indicated in Ref. 22 – Ref. 26. But the samples in these references can only represent a high-risk segment of the population and can not be compared to the situation in the rural setting of the region. Considering this issue might help experts to re-consider their modelling and data collection approach for the rural areas of the SSA region. Otherwise, the recommended policy directions will not lead to the desired outcome.
We appreciate the reviewer for providing a first-hand account of the situation in their country. Unfortunately, to our knowledge, few properly weighted indicators with unbiased sampling exists for cumulative infection rates in urban and rural areas, particularly in SSA countries so we rely primarily on serosurvey data to estimate relative urban-versus-rural infection rates. In figure figure 7, we show that the rural cumulative infections per capita is always lower in rural settings compared to urban ones but given the larger population in rural areas, the total number of cases may be larger in rural areas. Our results are therefore in line with the reviewer's observations and more recent serosurvey data from countries such as Senegal, South Africa and Ethiopia that are representative of the broader population. We have included these references in the results section (l. 282-284).

5. A minor but important comment: The vaccine type that is used in the study to simulate the impact is assumed to be highly efficacious and comparable to the Pfizer or Moderna mRNA vaccines (as indicated on page 9, line 140). However, it is known that the vaccine types that are distributed via COVAX are less efficacious than the one assumed in the model system. It is not clear why authors used such an assumption.
We thank the reviewer for this comment. We chose optimistic vaccine characteristics to highlight how despite having the best vaccines available, delayed delivery and not vaccine efficacy within an individual will be more detrimental to overall vaccine impact. Additionally, the effect of vaccines on severe cases and mortality can be approximated as a linear model so a less efficacious vaccine would lead to a commensurately lower effect on averting severe cases and mortality. We have included these points in the discussion. We also note that high efficacy vaccines are anticipated to come into the COVAX supply chain by 2022. This includes Novavax as well as dose donations of mRNA vaccines. We have now added a citation to the supply chain forecast in the manuscript to highlight this. Reference: https://www.gavi.org/sites/default/files/covid/covax/COVAX-Supply-Forecast.pdf
